# Synthesis of Novel Alkaline Protease-Incorporated Hybrid Nanoflowers (atP@hNFs) and Their Applications in the Laundry Detergent Industry

**DOI:** 10.1021/acsomega.5c05223

**Published:** 2025-09-25

**Authors:** Nihan Arabaci, Tuna Karaytug, Seyma Dadi, Ismail Ocsoy

**Affiliations:** 1 Department of Biology, Faculty of Arts and Sciences, Çukurova University, Adana 01250, Türkiye; 2 Department of Biology, Institute of Natural and Applied Sciences, Çukurova University, Adana 01250, Türkiye; 3 Department of Engineering Fundamental Sciences, Faculty of Engineering and Natural Sciences, Sivas University of Science and Technology, Sivas 58000, Türkiye; ∥ Department of Analytical Chemistry, Faculty of Pharmacy, Erciyes University, 38039 Kayseri, Türkiye

## Abstract

In recent years, enzyme-inorganic hybrid nanoflowers (hNFs) have received considerable attention due to their superior stability, enhanced catalytic activity, and reusability. In this study, we developed protease-hybrid nanoflowers (atP@hNFs) and evaluated their washing performance against protein-based stains in laundry detergents. For this purpose, the free protease (atP) from the *Bacillus cereus* strain TNP13 was produced and used to synthesize protease-hybrid nanoflowers (atP@hNFs). Subsequently, the atP@hNFs were characterized by various methods. Finally, both atP and atP@hNFs were utilized as bioadditives in laundry detergent formulations. The optimum pH and temperature values were determined to be pH 9.0 and 45 °C for free atP, and pH 10.0 and 55 °C for atP@hNFs, respectively. The pH stability of atP@hNFs showed a 23.3% increase compared to the free enzyme. Both free atP and atP@hNFs maintained stability across a temperature range of 20 to 75 °C. In the zymogram analysis of free atP, six activity bands with molecular weights of 123, 84, 71, 63, 57, and 49 kDa were detected. atP@hNFs showed significant increases in their activities compared to free atP in the presence of various metal ions such as Ca^2+^, Co^2+^, Mg^2+^, and Zn^2+^. While atP@hNFs maintained all their stability in H_2_O_2_, they were about 67% more stable in SDS than free atP. The washing performance analysis revealed that the stain removal capacity of atP@hNFs was superior to that of free atP in removing protein-based stains. These findings suggest that the atP@hNFs are particularly effective in cleaning protein-based stains and are a valuable candidate for use as a biological additive in various laundry detergents.

## Graphical abstract

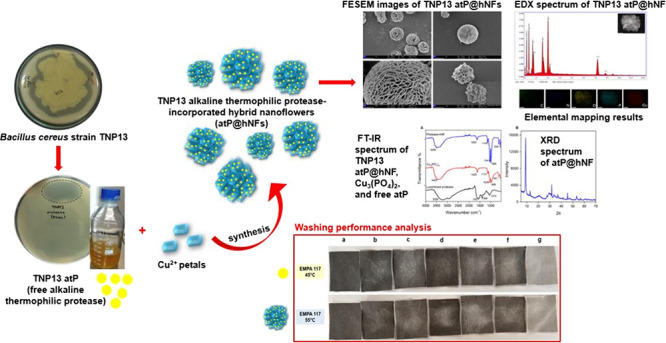

## Introduction

Proteases represent over 60% of the global enzyme market due to their broad industrial and biotechnological applications. Microbial proteases, especially alkaline proteases, are widely utilized in sectors including food, pharmaceuticals, cosmetics, textiles, leather, photography, waste treatment, and detergents, contributing nearly 40% of global enzyme sales. Alkaline proteases, in particular, are favored in detergent formulations due to their effectiveness in removing protein-based stains.^1^

Microorganisms are preferred in enzyme production due to their rapid growth, cost-effective cultivation, genetic manipulability, and high extracellular enzyme yield. *Bacillus* is among the most important microbial sources, producing extracellular enzymes with high catalytic efficiency and stability.^2^ Alkaline proteases (Subtilisins, EC 3.4.21.14) exhibit maximum activity in alkaline conditions, primarily at pH levels of 9.0–11.0, making them suitable for detergent applications.^3^ Industrial processes often involve harsh conditions such as extreme temperatures, pH variations, high salinity, and organic solvents. Therefore, enzymes used in industry must sustain their activity and stability under these conditions. In detergents, proteases are expected to maintain their activity and stability under washing conditions that include specific concentrations of salt, bleach, and surfactants at a pH range of 6.0–11.0 and a temperature range of 10–60 °C.^4^

Free enzymes typically suffer from instability, rapid loss of activity, and difficulty in recovery, which limit their applicability in industrial processes.^5^ Enzyme immobilization techniques have been developed to overcome these challenges to enhance stability, reusability, and activity.^6^ Since its discovery by Zare’s group in 2012, the organic–inorganic hybrid nanoflower has gained significant popularity in various scientific and technical fields as a novel method to enhance enzyme performance.^5,7,8^ The hNF is a 3D nanostructure that resembles a flower composed of organic constituents (enzymes, proteins, amino acids, biomolecules, etc.) and inorganic constituents (Cu^2+^, Ca^2+^, Mg^2+^, Fe^2+^, etc.).^9–11^ The formation of hNF relies on a coordination reaction between amide groups of organic components and metal ions in PBS.^7^ The synthesized hNF showed enhanced catalytic activity and stability compared to free enzymes, attributed to reduced mass transfer resistance, increased surface area, and favorable conformational changes in the enzyme structure.^12,13^ Hybrid nanostructures offer effective solutions to the limitations of enzyme sensitivity to environmental factors, low reproducibility, and purification difficulties. Enzymes immobilized on metal ions exhibit increased stability and activity, and are successfully applied in various industrial processes.^14,15^ Many studies have reported that enzyme catalytic activity and stability increase when the enzyme is synthesized in the hNF form. **Jamal and colleagues** ^16^ reported the synthesis of collagenase (Col) hNF with an encapsulation efficiency of 90% in the presence of Zn^2+^ ions. They stated that Col hNF retained more than 50% of its activity at 90 °C while the free enzyme maintained 17%. Additionally, Col hNF showed pH stability in a wide range. **Liu’s group** ^17^ synthesized thermophilic lipase hNFs from *Alcaligenes* sp. via biomimetic mineralization. These hNFs retained 80% of their activity after 4 weeks at room temperature, whereas the free enzyme lost most of its activity.

Nanoflower technology can be used to develop stable, active protease-hNFs for detergent formulations. These structures can significantly enhance the quality and stain removal performance of detergents. Obtaining protease-hNFs with high catalytic activity and stability will significantly contribute to production costs and the country’s economy. In this study, we describe the isolation and production of a thermophilic, detergent-compatible, and alkaline protease (free atP), the synthesis of Cu^2+^-based atP@hNF, the characterization of free atP and atP@hNF, and their applicability in laundry detergent formulations. Immobilization of TNP13 protease onto nanoflowers increased its stability under industrial conditions and improved the stain removal capacity on protein-based stains. Our findings suggest that atP@hNF is a promising candidate as a high-performance detergent additive, offering both functional and economic benefits.

## Experimental Section

### Isolation of a Protease-Producing Bacterial Strain

A 1-g soil sample from various chicken farms in Kayseri, Türkiye, was transferred into 50 mL of sterile LB medium.^18^ The suspension was subjected to heat shock at 80 °C for 10 min to eliminate vegetative strains that did not contain endospores.^19^ After this pretreatment, the samples were spread on LB agar (pH 9.0) and incubated at 37 °C overnight. Then, colonies with different morphological appearances were selected and cultured on LB agar (pH 9.0) as stock cultures.

The isolated strains were spread on SMA (pH 9.0) and incubated for 24 h at 37 °C. Transparent halo zones formed by the hydrolysis of skim milk showed the bacterial protease activity.^20^ Based on the “hydrolysis zone (mm)/colony diameter (mm)” ratio, the bacteria with the highest rate were selected as the best protease producers.^18^

### Identification of Bacteria

To identify the selected bacterial strain, biochemical tests, such as indole, hydrogen sulfide, motility, catalase, ability to use gelatin and casein, endospore, and Gram staining tests were performed.^21^ The strain was further identified by 16S rRNA gene sequencing at BM Laboratory Systems Inc., Ankara, Türkiye. The procedure used by the company is as follows: DNA isolation was performed using the “EurX GeneMATRIX Bacterial & Yeast DNA Isolation Kit (Poland)″. To determine the species, 27F (5′-AGAGTTTGATCMTGGCTCAG-3′) and 1492R (5′-TACGGYTACCTTGTTACGACTT-3′) universal primers were used to amplify the targeted region of the gene. In PCR analysis, the initial denaturation was performed at 95°C for 5 min. Thereafter, 30 cycles were performed under the following conditions: denaturation at 95°C for 45 s; annealing at 57°C for 45 s; extension at 72°C for 60 s. The final extension was completed at 72°C for 5 min. The MAGBIO ″HighPrep PCR Clean-up System″ (AC-60005) purification kit was used for single-band samples obtained in the PCR product purification stage. ABI 3730XL Sanger sequencer (Applied Biosystems, Foster City, CA) and BigDye Terminator v3.1 Cycle Sequencing Kit (Applied Biosystems, Foster City, CA) were used for Sanger Sequencing. The BLAST program searched for homologous sequences in the NCBI database. Evolutionary history was inferred using the Maximum Likelihood method and the Tamura-Nei model.^22^ MEGA X software was preferred during the evolutionary analyses.^23^

### Determination of Optimum Enzyme Production Conditions of Bacteria

The strain was cultured in skim milk-containing broth with pH values ranging from 6.0 to 12.0 and then incubated at 30–80 °C for 40 h. After incubation, the cultures were centrifuged at 7,500 rpm and 4 °C for 10 min, and the supernatant containing extracellular protease was used for standard enzyme activity analysis.^24^ As a result of the analyses, the optimum pH and temperature values at which the bacteria grow best and synthesize enzymes were determined.

### Enzyme Production

The method was modified from the work of **Jayakumar’s group** ^20^ For enzyme production, 1% (v/v) of the 24 h-old strain broth culture was inoculated into the skim milk-containing broth (pH 10.0) and incubated at 40 °C and 180 rpm for 5 days. After incubation, the culture was centrifuged at 7,500 rpm and 4 °C for 10 min. The obtained cell-free supernatant was used as a crude enzyme source for the following analysis.

### Lyophilization

The enzyme solution was lyophilized using a Christ lyophilizer at −85 °C under a 0.01 mbar vacuum. Subsequently, the crude protease (free atP) was obtained and immobilized with copper ions to form enzyme-hybrid nanoflower structures.

### Standard Enzyme Activity Analysis and Protein Estimation

For activity determination, the free atP and atP@hNF enzymes and 0.2 M glycine-NaOH buffer solutions containing 1% casein at the optimal pHs (pH 9.0 for free atP and pH 10.0 for atP@hNF) were mixed in equal volumes and incubated in a water bath at the optimal temperatures (45 °C for free atP and 55 °C for atP@hNF) for 60 min, separately. The reactions were terminated by adding an equal volume of 10% TCA solution to the enzyme–substrate mixture. The mixtures were then centrifuged at 10,000 rpm for 10 min at 4 °C. Subsequently, 0.5 mL of the supernatant, containing the reaction products of the enzyme–substrate reaction, was transferred to clean tubes, followed by the addition of 2.5 mL of 0.5 M Na_2_CO_3_ solution to each tube. Then, 0.5 mL of Folin-Ciocalteu’s phenol reagent (diluted 1:2 with distilled water) was added to the mixtures. After incubation at room temperature for 30 min, absorbances were measured at 660 nm against a blank.^24^

The Bradford method was used to determine protein quantity by generating a BSA calibration curve.^25^ One unit of enzyme activity was defined as the amount of enzyme that hydrolyzes casein under standard reaction conditions, releasing 1 μmol of tyrosine per minute. A tyrosine calibration curve was prepared as a standard to calculate protease activity, and the equation obtained from the curve.^26^

### Molecular Weight Determination and Zymogram Analysis

Zymogram analysis was performed by SDS-PAGE^27^ using a 10% polyacrylamide gel containing 1% casein as the substrate. The enzyme sample was mixed with loading buffer at a 1:5 ratio. Then, electrophoresis was performed at a constant current of 15 mA by loading the samples into the wells of the gel. After electrophoresis, the gel was incubated in 200 mM glycine-NaOH buffer containing 2.5% Triton X-100 at room temperature for 30 min to remove SDS. Then the gel was kept in a 200 mM glycine-NaOH buffer containing 1% casein for 60 min. After incubation, the gel was stained in a Coomassie Brilliant Blue R250 (CBB R250) solution for 2 h to make the enzyme activity bands visible. Light-colored regions indicating the proteolytic activity were observed on the gel.^28^ The proteolytic activity bands were calculated by comparison with the marker to determine the molecular weight. The marker protein (Pre-Stained Ladder, 10–180 kDa, Thermo Fisher Scientific) was loaded into the well of a substrate-free gel. A separate SDS-PAGE was performed under the same conditions. After electrophoresis, the gel was stained using CBB R250 dye solution at room temperature for 2 h. The gel was washed in dye removal solution overnight to remove excess dye remaining in the gel. Finally, the bands were determined and compared with the standard marker bands.^29^

### Effects of pH on Enzyme

The optimum pH of the enzyme was determined using sodium-phosphate (pH 6.0–8.0), glycine-NaOH (pH 9.0–10.0), sodium bicarbonate (pH 11.0), and Na_2_HPO_4_–Na_3_PO_4_ (pH 12.0) buffers containing 1% casein.^30^ For activity determination, enzyme and buffer solutions at different pHs were mixed in equal volumes and incubated at 40 °C for 60 min. Then, the standard enzyme activity was measured as described under the heading ″Standard Enzyme Activity Analysis and Protein Estimation″.^31^

To determine pH stability, substrate-free pH buffers (pH 6.0–12.0) and the enzyme were mixed in a 1:1 ratio and incubated at room temperature for 60 min. After preincubation, residual enzyme activities were analyzed using optimum pH buffers containing 1% casein, following the standard enzyme activity analysis method described in the ″*Standard Enzyme Activity Analysis and Protein Estimation*″ section.^32^ These activities were compared with a control (100%) (initial enzyme activity measured under optimum pH).

### Effects of Temperature on Enzyme

To determine the optimum temperature, free atP and atP@hNF enzymes were separately mixed in equal volumes with casein solutions buffered at pH 9.0 (for free atP) or pH 10.0 (for atP@hNF), and incubated for 60 min at temperatures ranging from 20 to 75 °C. Enzyme activities were measured using the method described in the “*Standard Enzyme Activity Analysis and Protein Estimation*” section.^3^

For thermal stability analysis, the enzymes were preincubated at temperatures ranging from 20 to 75 °C for 60 min. After preincubation, the residual enzyme activities were determined using the same method described in the section mentioned above. Finally, these activities were compared with a control (100%) (the activity of the enzyme that is not exposed to any temperature).^31^

### Effects of Metal Ions, Inhibitors/Chelators, and Surfactants/Oxidizing Agents on Enzyme Activity

The enzyme was mixed with various chemicals, including metal ions [CaCl_2_, CoCl_2_, CuCl_2_, MgCl_2_, MnCl_2_, NiCl_2_, ZnCl_2_, (5 and 10 mM)], inhibitors and chelators [PMSF (1 and 5 mM), EDTA (1 and 5 mM), urea (5 and 10 mM), β-mercaptoethanol (0.01% and 0.05%), TLCK (1 and 2 mM), 1,10-phenanthroline (1 and 5 mM), iodoacetamide (1 and 5 mM)], and surfactants/oxidizing agents [SDS (0.1% and 0.5%), Triton X-100, Tween 20, Tween 80, CTAB, and H_2_O_2_ (0.5% and 1%)]. The mixtures were preincubated at room temperature for 60 min.^32,33^ Then the samples were taken from each reaction mixture, and standard enzyme activity was assayed. The residual activities (%) were calculated by comparing with the control (100%), which contains no chemicals. The experiments were performed in triplicate.

### Effect of NaCl on Enzyme Activity

The enzyme was mixed with NaCl solutions at concentrations of 1, 5, 10, 15, and 20%. Then the mixtures were preincubated at room temperature for 60 min. Samples were taken from each reaction mixture, and standard enzyme activity was measured. The residual activities (%) were calculated by comparing with the control (100%) (without NaCl).^3^ The experiments were repeated three times.

### Determination of Hydrolysis Products by TLC

The TLC method was adapted from **Guleria and colleagues’** ^34^ work. Hydrolysis products (15 μL) of the enzyme–substrate reaction were analyzed using chromatography plates (TLC F_254_ plates, 20 cm × 20 cm, Merck, Darmstadt, Germany). Standard amino acids prepared at 1% concentration, including l-Tyrosine, l-Histidine, l-Cysteine, l-Glycine, l-Arginine, l-Alanine, l-Aspartic Acid, l-Lysine, and l-Serine, were used as markers (1 μL). Butanol-acetic acid-distilled water (3:1:1, v/v/v) solution was used as the mobile phase. The plate on which the samples were loaded was immersed in the mobile phase solution, and the molecules were allowed to migrate in silica gel for approximately 12 h. At the end of the migration, a dye solution (acetone solution containing ninhydrin) was sprayed on the plate to visualize the products. Then, the plates were heated at 90 °C for 20 min. The reaction products were identified by comparison with the amino acid markers.

### Synthesis of Protease-Hybrid Nanoflowers

The procedure in the literature was followed in the synthesis of atP@hNFs.^35^ 0.02 mg/mL of lyophilized free atP solution was mixed with 10 mM phosphate buffer (pH 7.2) on a magnetic stirrer at room temperature for 15 min. Then, CuSO_4_ solution (120 mM, 6.67 mL) was added to the mixture and incubated at room temperature for 3 days. The blue precipitates formed (hNF structures) were collected by centrifugation, washed with deionized water, dried at 37 °C, and stored for subsequent analyses. Specifically, the exact mass-to-mass ratio of the enzyme and copper was 0.45.

### Calculation of Encapsulation Ratio

The Bradford method was used to calculate the encapsulation ratio of the protease in the hNF structure.^25,36^ BSA solutions were prepared at 0.001 to 0.05 mg/mL concentrations to generate the standard curve. One mL of each BSA concentration was mixed with 5 mL of Bradford reagent. After incubation at room temperature for 20 min, the absorbance was measured at 595 nm. A standard curve was plotted based on the absorbance values corresponding to the concentrations. To calculate the encapsulation ratio, 1 mL of the supernatant solution (separated after synthesis) was mixed with 5 mL of Bradford reagent and incubated at room temperature for 20 min. The absorbance was measured at 595 nm, and the concentration was calculated based on the standard curve obtained. The following formula was used to calculate the encapsulation ratio:

$$\%E=\frac{\mathrm{Ct}-\mathrm{Cs}}{\mathrm{Ct}}\times 100$$

(Ct: Total enzyme amount, Cs: Enzyme amount remaining in the supernatant).

### Characterization of Synthesized Hybrid Nanoflower Structures

FESEM (Zeiss Gemini 500) was used for imaging the morphology of atP@hNFs. EDX was used for elemental analysis of hNF. The crystal structures of hNFs were determined with XRD (Panalytical Empyrean), and the functional groups analysis of hNFs was performed using FT-IR (Thermo Scientific Nicolet 6700). Absorbance measurements were carried out with a UV–vis Spectrometer (Shimadzu, UV1900).

### Determination of Activities of atP@hNFs

The procedures described in the “*Standard Enzyme Activity Analysis and Protein Estimation*” section were also applied to determine the activity of the atP@hNFs. All parameters related to enzyme characterization applied with free atP were also performed with atP@hNFs using the same methods. atP@hNFs were sonicated before being added to the reaction mixtures described in the methods.

### Detergent Compatibility Analyses of Free atP and atP@hNF

To assess the detergent compatibility, five commercial liquid laundry detergents [Ariel (Procter & Gamble), Bingo (Hayat Kimya), OMO (Unilever), Persil (Henkel), Perwoll (Henkel)] were boiled for 2 h to inactivate the endogenous enzymes in their composition. Detergents were added to the reaction medium at a final concentration of 1% and incubated with atP@hNF at room temperature for 60 min. After taking samples from each reaction mixture, the residual activities were calculated using standard enzyme activity assays and compared with the control (100%), which contained no detergent.^3,28^

### Washing Performance Analysis of Free atP and atP@hNF

For the washing performance test, standardized stained fabrics coded EMPA 117 (blood/milk stain on polyester/cotton (65/35) fabric), CFT CS-38 (egg stain on cotton fabric), and CFT CS-03 (milk chocolate stain on cotton fabric) were used. To evaluate the washing performances of free atP and atP@hNF, the analyses were simultaneously performed with the commercial powder protease (Novozyme Savinase, Batch no: 015–11013, enzyme activity: 5.89 KNPU­(S)/g, country of origin: Denmark). The following mixtures were prepared in beakers to analyze the washing performances of the enzymes:

1. Tap water (control).
2. Commercial protease + tap water (control),
3. Free atP + tap water,
4. atP@hNF + tap water,
5. 1% liquid detergent + tap water (control),
6. Commercial protease + liquid detergent + tap water (control),
7. Free atP + liquid detergent + tap water,
8. atP@hNF + liquid detergent + tap water

Precut fabric pieces (4 cm × 4 cm) were placed in beakers and incubated separately for 60 min with free atP at 45 °C and atP@hNF at 55 °C at 100 rpm. Washing analyses with commercial protease were performed at the optimum temperatures of free atP and atP@hNF. After washing, the fabrics were rinsed with tap water and air-dried at room temperature. Then, the stain removal abilities of free atP and atP@hNF were visually evaluated and compared with each other and the control groups.^37,38^

## Results and Discussion

### Strain Isolation, Protease Activity, and Identification

The strain, isolated as described in “*Experimental Section*” and determined to exhibit protease activity **(** Figure 1A**),** was identified using 16S rRNA analysis and molecular identification using the Sanger Dideoxy Sequencing technique. The strain was identified as “*Bacillus cereus* strain TNP13” and registered in the NCBI GenBank database (https://www.ncbi.nlm.nih.gov/nuccore/OL677431.1). The phylogenetic tree was constructed as shown in Figure 1(B).

**Figure 1:**
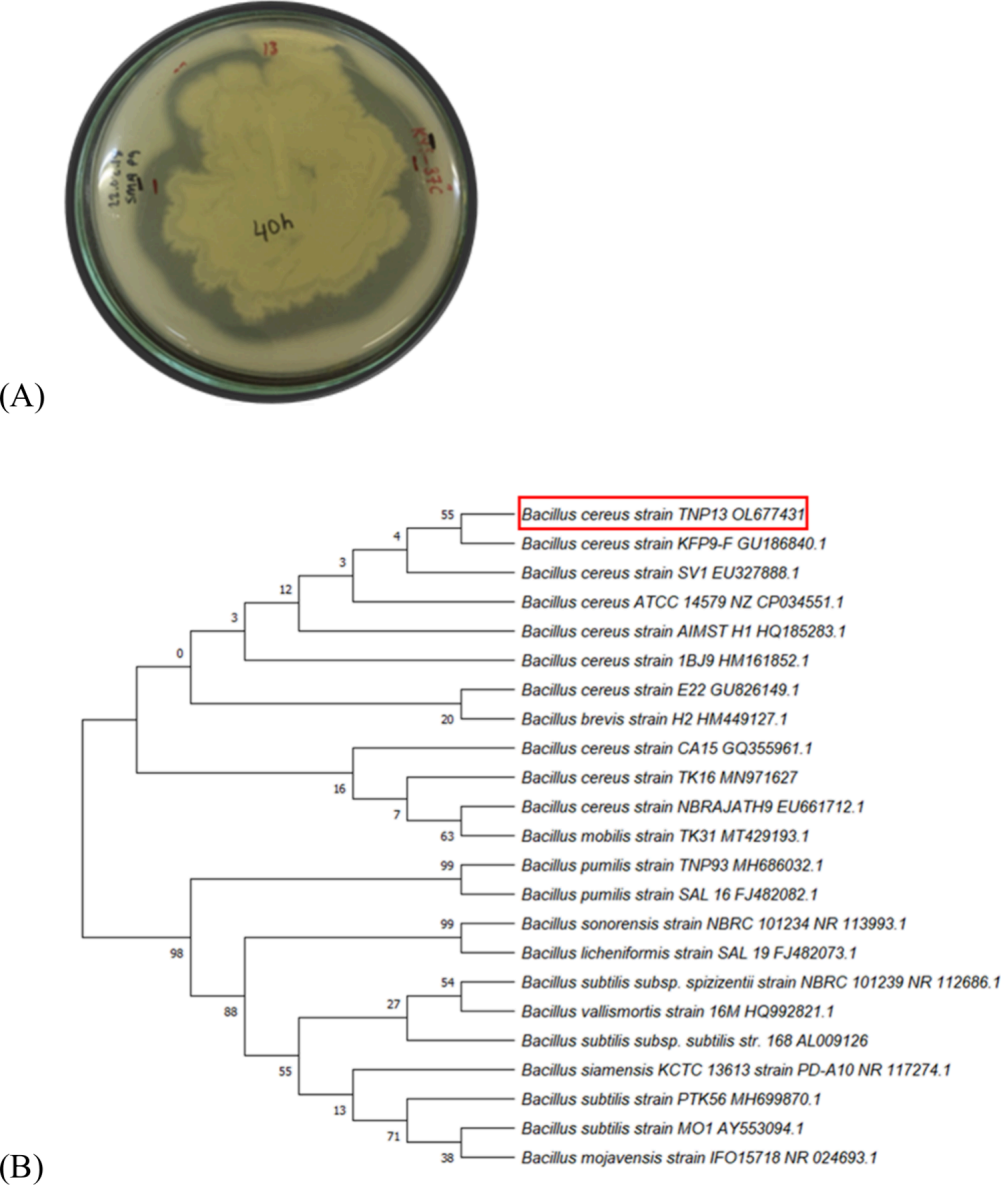
*Bacillus cereus* strain TNP13. (A) Protease activity on SMA and (B) phylogenetic tree.

### Protease Production and Lyophilization

The optimum temperature at which the bacterial strain we isolated grew best and synthesized protease was determined as 40 °C, and the optimum pH was 10.0. After the strain was grown in broth culture medium containing skim milk for protease production, the supernatant obtained was collected to be used as a raw enzyme source in all studies **(** Figure 2A**)**. The supernatant was lyophilized as described in the “*Lyophilization*” section.

**Figure 2:**
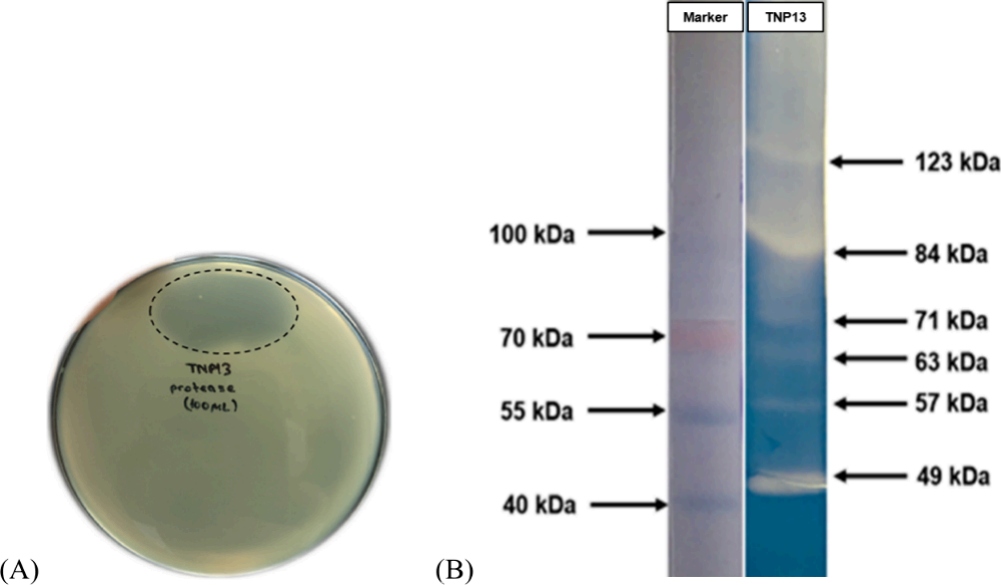
TNP13 protease (free atP). (A) Hydrolysis zone on SMA. (B) Zymogram analysis.

### Molecular Weight of Protease and Zymogram Analysis

Zymography of the protease produced by the *B. cereus* strain TNP13 was performed with a 10% polyacrylamide gel containing 0.5% (w/v) skim milk under non-reducing conditions. The transparent regions of proteolytic hydrolysis observed in the dark blue zymogram gel were compared with the protein marker bands in the gel of SDS-PAGE performed under identical conditions. According to SDS-PAGE and Zymogram analysis, six proteolytic activity bands of TNP13 protease with molecular weights of 123, 84, 71, 63, 57, and 49 kDa were detected, respectively **(** Figure 2B**).** In comparison, various purified proteases with molecular weights of 28, 34.6, and 42 kDa have been reported in the literature.^39–41^

### Protein and Unit Amount

A BSA standard curve was prepared to determine the protein concentration (mg/mL). The slope of the curve was 0.0017, with an R^2^ value of 0.9929. According to the protein standard curve, the protein concentration of TNP13 free atP was determined to be 19.42 mg/mL, while the protein concentration of atP@hNF was calculated to be 165 mg/mL. The tyrosine standard curve was prepared to determine enzyme activity (U), and the equation was obtained from the graph. The slope obtained, based on the R^2^ = 0.9997 value and the equation in the curve, is 1.6805. Accordingly, the activities of TNP13 free atP and atP@hNF were calculated as 4.5 U/mL and 38.5 U/mL, respectively.

### Synthesis and Characterization of atP@hNFs

The hNF structures were synthesized from the protease of *B. cereus* strain TNP13 in phosphate buffer by single-step biomineralization. In the synthesis mechanism, nitrogen atoms in amine or amide groups of the protease can form a complex with Cu^2+^ ions via a coordination reaction. This complex binds the hNF petals together. The morphologies of the synthesized atP@hNFs were analyzed with FESEM. Flower-shaped and spherical morphologies were obtained from the synthesis **(** Figure 3A**).** As seen in SEM images, flower-like morphologies were formed by the adhesion of the petals to each other.

**Figure 3:**
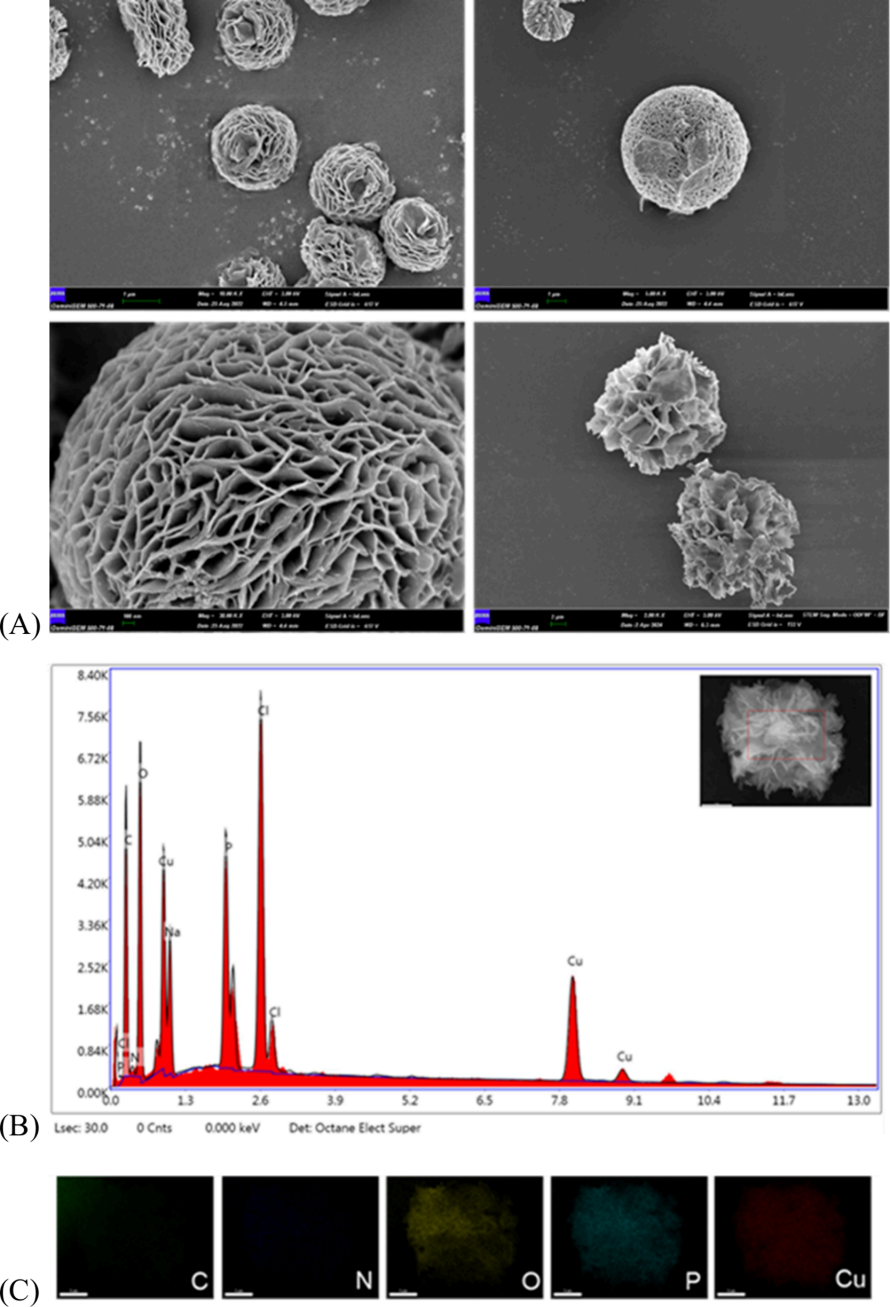
(A) FESEM images of TNP13 atP@hNFs, (B) EDX spectrum of TNP13 atP@hNF, and (C) elemental mapping results.

According to the encapsulation ratio calculated based on the amount of protein remaining in the supernatant of the hNF synthesis solution, 0.02 mg/mL of TNP13 free protease (atP) was encapsulated into Cu_3_(PO_4_)_2_ crystals with an efficiency of 90.2%. In the literature, various encapsulation efficiencies of hNFs synthesized using different enzymes have been reported. **Nadar and colleagues** ^42^ reported that glucoamylase solution (0.05 mg/mL) was encapsulated into glucoamylase-hNF at a rate of 85.25%, and they obtained flower-like structures after 24 h of incubation. **Altınkaynak and co-workers** ^15^ calculated an encapsulation ratio of 94% in lactoperoxidase-hNF synthesis. In another study, when cholesterol-oxidase solution (0.05 mg/mL) and 1.6 mM CuSO_4_ were used, the encapsulation ratio was 69.7%, and the morphology of the hNF obtained was spherical and flower-shaped.^43^ **Gulmez and co-workers** ^44^ synthesized proteinase K–Cu^2+^ hNF with varying concentrations of proteinase K (0.01, 0.02, 0.05, and 0.1 mg/mL). According to the SEM image, the morphology of hNF synthesized at 0.02 mg/mL was more uniform than that of the others, with an encapsulation ratio of 98%. In another study, trypsin-Zn_3_(PO_4_)_2_-hNF structures were influenced by the concentration of trypsin. As the amount of trypsin increased from 0.01 to 0.25 g, the particle size of hNF decreased, the petals became thinner, and the hNF morphology changed significantly.**^45^** The characterization findings of atP@hNF structures align with those reported in similar studies in the literature.

The elemental composition of the synthesized atP@hNF structure was determined by SEM-EDX analysis. The EDX spectrum of the C, O, P, and Cu elements found in the hNF structure is presented in Figure 3(B). The peaks corresponding to C and N come from protease, while the peaks of the Cu and P elements belong to Cu_3_(PO_4_)_2_. The O element is found in both structures. The elemental mapping results demonstrate that the C, O, P, and Cu elements found in the hNF structure are homogeneously distributed (Figure 3C**)**.

Protein analysis was performed using the Bradford method to calculate the enzyme’s attachment rate to Cu_3_(PO_4_)_2_ crystals. The BSA standard curve used is shown in Figure 4.

**Figure 4:**
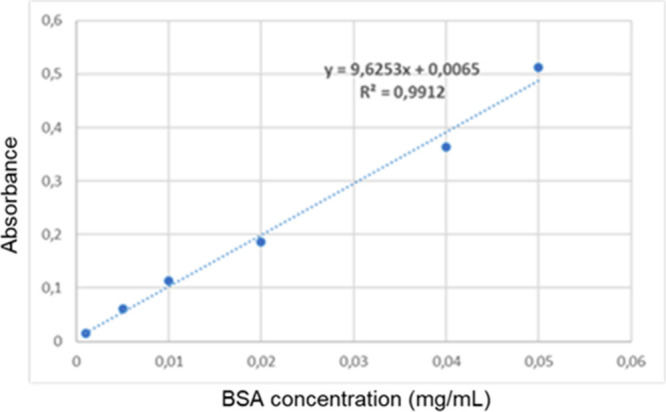
Standard BSA curve.

The chemical structure of TNP13 atP@hNF was determined using FT-IR spectroscopy. In the spectrum of Cu_3_(PO_4_)_2_ shown in Figure 5(A), the peak at 621 cm^–1^ represents the bending vibration of P–O groups, while the peaks at 969 cm^–1^ and 1029 cm^–1^ correspond to the stretching vibrations of P–O and P = O, respectively.^45^ The peaks associated with the bending and stretching vibrations of P–O and P = O appear at 628 cm^–1^, 988 cm^–1^, and 1041 cm^–1^ in the hNF spectrum. These peaks confirm the presence of Cu_3_(PO_4_)_2_ crystals in the synthesized hNF structure. The XRD spectra of the synthesized hNF and the reference standard (PDF 22–0548) are presented in Figure 5 **(B, C).** Since the diffraction peaks of atP@hNF align with the degree values of the diffraction peaks of the standard, this indicates that the crystal structure of hNF is compatible with that of Cu_3_(PO_4_)_2_.

**Figure 5:**
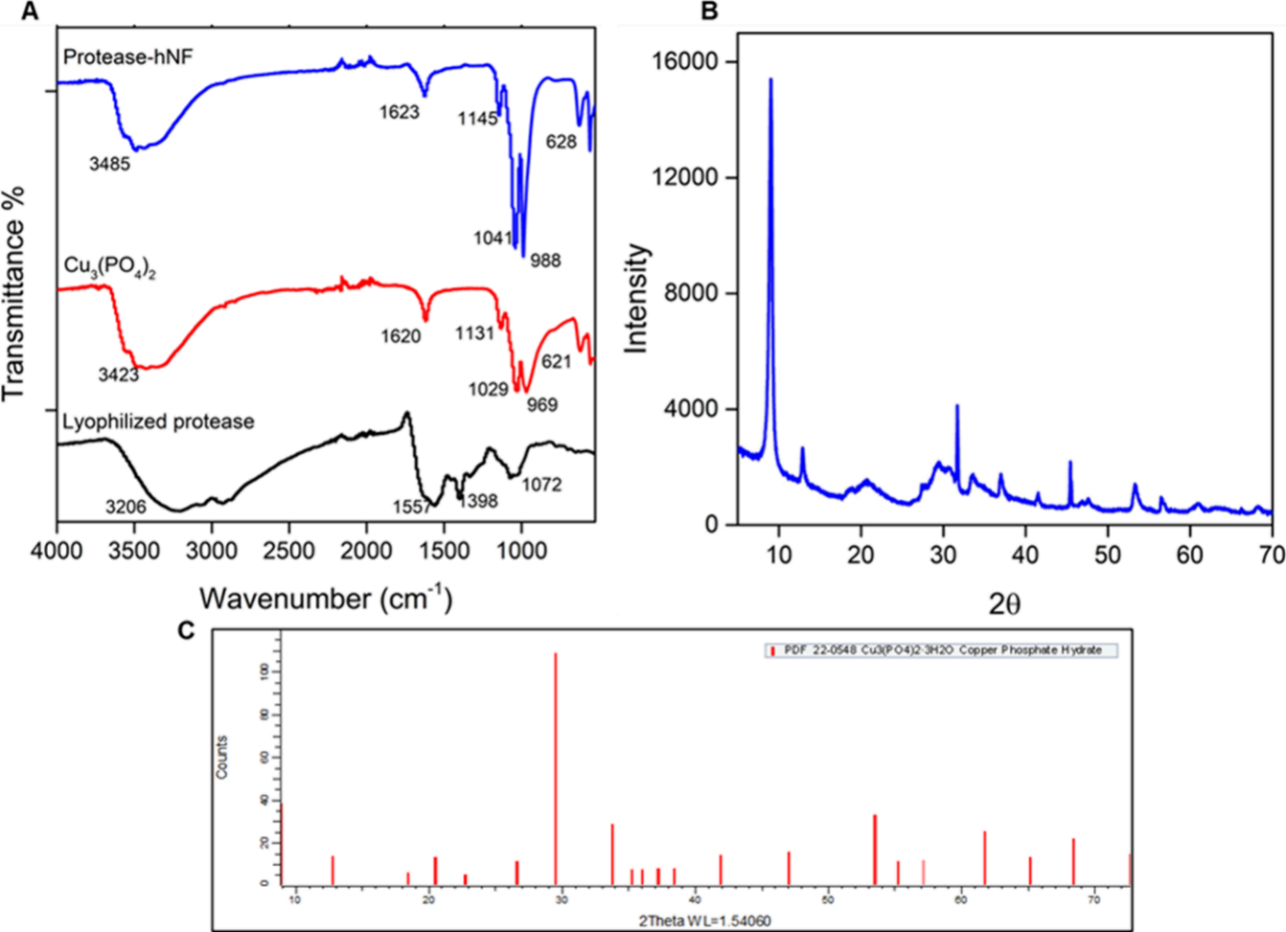
(A) FT-IR spectrum of TNP13 atP@hNF, Cu_3_(PO_4_)_2_, and free atP, (B) XRD spectrum of atP@hNF, and­(C) XRD spectrum of standard card (PDF 22–0548).

### Effects of pH and Temperature

Conventional immobilization methods often cause enzyme denaturation and loss of catalytic activity. However, enzymes immobilized in nanoflower structures maintain their stability under elevated temperature and pH conditions.^46^ In our study, the optimum activities of the free atP isolated from the TNP13 strain and the synthesized atP@hNF were determined as pH 9.0 and 10.0, respectively **(** Figure 6A**)**. The optimum activity pH of the free atP, which has an alkaline character, increased in the nanoflower structure after nanoparticle synthesis. The shift in pH optima can be explained by the changes in the enzyme’s active site due to covalent immobilization during the synthesis of atP@hNF compared to the free enzyme.^47^ Similar observations have been reported in the literature. **Ibrahim and colleagues** ^47^ reported that the optimum pH of AK-protease shifted from 10.0 to 10.5 upon immobilization. **Gulmez and co-workers** ^44^ reported that the optimum pHs of free proteinase K and proteinase K-hNF were 10.0 and 11.0, respectively. **Kumari’s group** ^48^ determined that the pH of free atP showed optimum activity at 9.0. The protease immobilized in crystalline mesoporous zeolite (Nano ZSM-5) also showed its optimum activity at pH 10.0.

**Figure 6:**
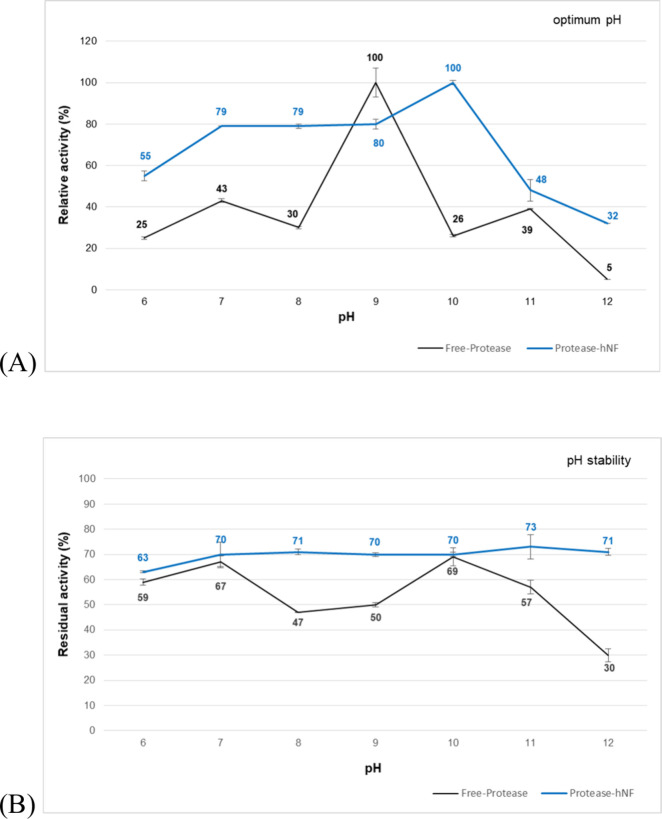
(A) Optimum pHs and (B) pH stabilities of TNP13 free atP and atP@hNF. The activity of the control was used in calculating the stability values.

The alkaline TNP13 protease exhibited an average stability of approximately 60% within the pH range of 9.0–11.0 **(** Figure 6B**)**. After synthesis of free atP in nanoflower form, the stability of atP@hNF was about 74% in the pH range of 9.0–11.0. The pH stability of the TNP13 free atP increased by 23.3% in the hNF form. With nanoparticle synthesis, the enzyme mobility decreases, and the amount of binding with metal ions increases. This decreases conformational modifications caused by pH changes, expanding the pH range in which the enzyme maintains stability. Therefore, the immobilized enzyme exhibited a better catalytic performance than its free form.^14,16^ Similarly, **Zhang and co-workers** ^49^ reported that the free alkaline protease obtained from *B. licheniformis* showed lower stability in the pH range of 7.0 to 9.0 compared to its nanoflower form. **Jamal’s team** ^16^ also found that the immobilized collagenase maintained its stability at higher rates within the pH range of 4.0 to 9.0 compared to free collagenase.

TNP13 free atP and atP@hNF exhibited optimum catalytic activities at 45 and 55 °C, respectively **(** Figure 7A**).** The temperature value at which the thermophilic free protease showed optimum activity increased when the protease was synthesized as a nanoflower structure. In addition, the atP@hNF showed higher relative activity (%) at all temperature points. This increase in the optimum temperature activity of the immobilized enzyme can be attributed to improved conformational stability due to covalent immobilization.^47^ **Ibrahim and colleagues** ^47^ reported that the optimum temperature of free AK-R protease was 60 °C, which increased to 65 °C after immobilization. **Badoei-Dalfard’s group** ^14^ declared that the free collagenase from *B. subtilis* COL3 exhibited optimum activity at 50 °C. When the enzyme was synthesized as magnetic cross-linked collagenase metal hybrid nanoflower (mcCNF), its optimum temperature was increased to 55 °C. **Jamal and group** ^16^ reported that the optimum temperatures of free collagenase and its Col-Zn-hNF form were 50 °C.

**Figure 7:**
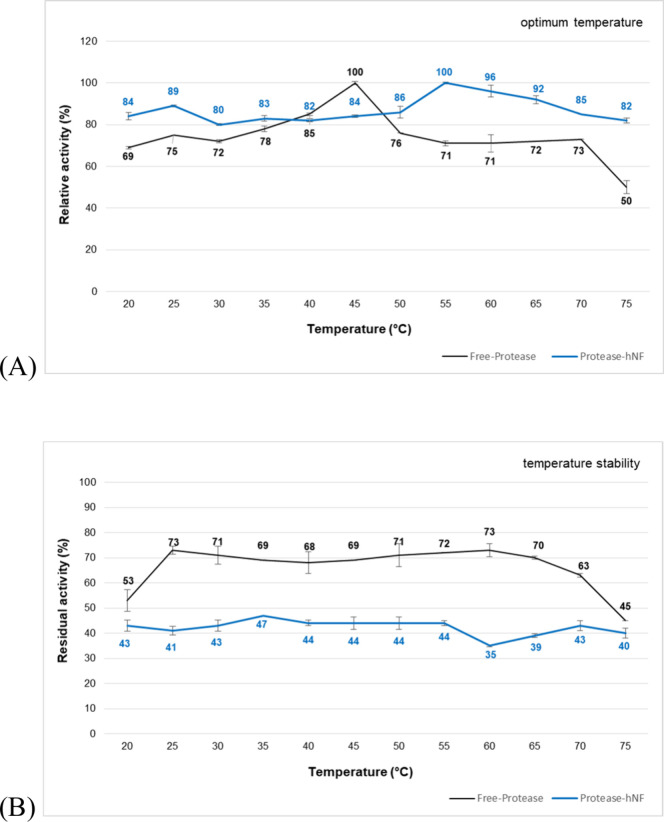
(A) Optimum temperatures and (B) temperature stabilities of TNP13 free atP and atP@hNF. The activity of the control was used in calculating the stability values.

The thermophilic free TNP13 protease retained ∼ 72% of its activity between 40 and 60 °C **(** Figure 7B**).** After nanoparticle synthesis, the atP@hNF retained ∼ 43% of its activity in the same temperature range. The thermal stability of free atP was higher than that of the atP@hNF. A similar result has been observed in several studies in the literature. **Zhu and collaborators** ^50^ found that the thermal stability of the AY-10@AXH-HNF structure synthesized with endopeptidase and exopeptidase enzymes was lower than that of the free enzymes. This decrease was attributed to the biomineralization conditions during the synthesis, which may have affected enzyme thermostability by altering the protease structure. At high temperatures, copper particles cannot self-assemble to form nanoflower structures, resulting in sparse petal formation. It has been reported that the formation of Cu_3_(PO_4_)_2_ is an exothermic reaction, and high temperatures make this formation difficult. Consequently, enzyme immobilization within the hybrid nanoflower structure becomes less efficient, and its thermostability is adversely affected.^51^

In this context, this finding aligns with our results. Despite this, certain studies in the literature generally report that the temperature stability of the immobilized enzyme is higher than that of the free enzyme.^14,47,48^

### Effect of Metal Ions, Inhibitors/Chelators, and Surfactants/Oxidizing Agents on Enzyme Activity

Stability results of TNP13 free atP and atP@hNF in the presence of various chemicals are given in Table 1. In various industrial applications, particularly in the detergent industry, metal ions in the process environment can affect enzyme reactions differently. They can act as cofactors to activate or inhibit the enzyme’s function.^52^ They can also influence the activity by generating or neutralizing electrophilic or nucleophilic groups via electron exchange. Additionally, they can activate the enzyme by forming a bridge between the enzyme and the substrate and stabilizing the three-dimensional catalytic structures of enzymes.^41^ Alkali metals such as Na^+^, K^+^, Li^+^, Mg^2+^, Ca^2+^, and Ba^2+^ typically bind to enzymes through ionic bonds, protecting against thermal denaturation.^20,53^ In this study, the activity of atP@hNF increased by 81.58%, 139.13%, and 66.67%, respectively, compared to free atP in the presence of 10 mM Ca^2+^, Co^2+^, and Mg^2+^, while the activity increased by 63.41% in the presence of 5 mM Zn^2+^. These significant increases indicate that immobilization positively affects enzyme activity. In particular, Cu^2+^ ions caused a strong inhibition (over 70%) of atP@hNF activity. While studies on the effects of metal ions on immobilized enzymes in general are available, there are limited data specifically comparing free and immobilized proteases. Existing studies comparing free enzyme and immobilized enzyme report the effects of metal ions on the activities of various proteins or the stability of enzymes other than proteases.^54,55^ The effects of specific inhibitors were tested to better understand the nature of the enzyme. The inhibition of TNP13 free atP by PMSF, TLCK, EDTA, and 1,10-phenanthroline indicated that the enzyme is a serine metalloprotease. PMSF and 1,10-phenanthroline inhibit proteases by sulfonating essential serine residues in the active site.^33^ No studies have been found comparing the effects of inhibitors on the activities of free and immobilized proteases. Available comparative studies report the effects of inhibitory substances on the activities of different proteins or the stability of enzymes other than proteases.^55^ In our research, TNP13 atP@hNF generally showed higher stability than free atP when exposed to cationic detergents. While atP@hNF retained all its stability when exposed to H_2_O_2_, an oxidizing agent, it showed approximately 67% more stability than free atP with SDS, an anionic detergent. Both atP@hNF and free atP remained stable with Tween 20 and Tween 80, but atP@hNF maintained its stability at a rate 25% higher than free atP at 0.5%. In the presence of 1% Triton X-100, no difference in stability was noted between the two enzyme forms. **Badoei-dalfard and co-workers** ^14^ treated free collagenase and collagenase nanoflower structures with various surfactants, including Tween 20, H_2_O_2_, Tween 80, CTAB, Triton X-100, and SDS. The activity of free collagenase decreased by approximately 27%, 33%, and 27% in SDS, CTAB, and H_2_O_2_, respectively, while nanoflower structures decreased by approximately 13%, 15%, and 4%, respectively. When **Jamal and colleagues** ^16^ evaluated the activities of free collagenase and collagenase-hNF with Tween 20, Tween 80, Triton X-100, SDS, and CTAB, they found that Col-Zn-hNFs showed better activity than free enzymes with all surfactants.

**Table 1:** Effects of Various Chemicals on Free atP and atP@hNF

**chemicals**	**free atP**		**atP@hNF**		
**control**		**residual activity (%)**		**residual activity (%)**	
**metal ions**	concentration (mM, %)	**100**	concentration (mM, %)	**100**	**change in residual activity (↑%–↓%)**
CaCl_2_	5 mM	57 ± 0.86	5 mM	71 ± 2.53	**↑** 24.56
	10 mM	38 ± 1.30	10 mM	69 ± 1.20	↑ 81.58
CoCl_2_	5 mM	72 ± 0	5 mM	53 ± 2.92	↓ 26.39
	10 mM	23 ± 2.26	10 mM	55 ± 2.00	**↑** 139.13
CuCl_2_	5 mM	160 ± 9.24	5 mM	26 ± 1.39	↓ 83.75
	10 mM	64 ± 0	10 mM	18 ± 1.60	↓ 71.88
MgCl_2_	5 mM	56 ± 0	5 mM	72 ± 2.31	**↑** 28.57
	10 mM	36 ± 0	10 mM	60 ± 1.04	**↑** 66.67
NiCl_2_	5 mM	60 ± 2.36	5 mM	65 ± 1.09	↑ 8.33
	10 mM	63 ± 0	10 mM	53 ± 1.82	↓ 15.87
ZnCl_2_	5 mM	41 ± 2.80	5 mM	67 ± 1.78	↑ 63.41
	10 mM	56 ± 4.10	10 mM	53 ± 2.59	↓ 5.36
**Inhibitors/Chelators**					
PMSF	1 mM	23 ± 2.40	1 mM	60 ± 1.40	↑ 160.87
	5 mM	24 ± 2.80	5 mM	51 ± 2.27	↑ 112.5
TLCK	1 mM	98 ± 0.39	1 mM	55 ± 3.10	↓ 43.88
	2 mM	35 ± 1.18	2 mM	59 ± 1.73	↑ 68.57
1,10-phenanthroline	1 mM	95 ± 0.32	1 mM	64 ± 0.41	↓ 32.63
	5 mM	58 ± 1.02	5 mM	49 ± 0.75	↓ 15.52
iodoacetamide	1 mM	53 ± 0	1 mM	60 ± 1.02	↑ 13.21
	5 mM	40 ± 0	5 mM	57 ± 2.63	↑ 42.50
EDTA	1 mM	51 ± 7.10	1 mM	67 ± 1.92	↑ 31.37
	5 mM	51 ± 6.70	5 mM	55 ± 3.10	↑ 7.84
β-mercaptoethanol	0.01%	49 ± 1.77	0.01%	70 ± 1.50	↑ 42.86
	0.05%	53 ± 0.76	0.05%	32 ± 5.30	↓ 39.62
urea	5 mM	89 ± 0	5 mM	65 ± 2.20	↓ 29.97
	10 mM	64 ± 0.66	10 mM	67 ± 3.68	↑ 4.69
**Surfactants/Oxidizing Agents**					
Tween 20	0.5%	79 ± 1.10	0.5%	78 ± 0.41	↓ 1.27
	1%	93 ± 0.36	1%	85 ± 1.54	↓ 8.60
Tween 80	0.5%	70 ± 4.10	0.5%	77 ± 3.64	↑ 10
	1%	88 ± 9.5	1%	86 ± 1.54	↓ 2.27
TritonX-100	0.5%	64 ± 8.75	0.5%	80 ± 11.30	↑ 25
	1%	78 ± 0.18	1%	76 ± 3.61	↓ 2.56
CTAB	0.5%	65 ± 6.61	0.5%	47 ± 2.70	↓ 27.70
	1%	64 ± 3.57	1%	48 ± 2.60	↓ 25
H_2_O_2_	0.5%	92 ± 1.43	0.5%	115 ± 5.20	↑ 25
SDS	0.5%	75 ± 5.89	0.5%	125 ± 1.02	↑ 66.67

### Effect of NaCl on Enzyme Activities

The presence of salt in the enzyme–substrate reaction medium increases the osmotic pressure. This increase can alter the properties of the solution containing the enzyme, potentially affecting its stability and causing the enzyme to denature.^56^ In our study, when TNP13 free atP was exposed to a 1% NaCl concentration, a significant loss of stability (72%) occurred **(** Figure 8 **).** Conversely, when the atP@hNF was treated with various NaCl concentrations, the enzyme’s stability improved, maintaining over 70% of its activity at NaCl concentrations of 1–10%. The hNF structure formed by nanoparticle synthesis ensured that the stability of the protease was maintained. TNP13 atP@hNF was halotolerant and exhibited greater salt tolerance than the free atP. Therefore, halotolerant atP@hNF has a high potential for use in laundry detergents, especially in laundry processes performed in groundwater with high salt content.^57^ **Ibrahim’s group** ^47^ evaluated the activities of free and immobilized AK-R alkaline proteases in a wide range of NaCl concentrations (up to 25%, w/v), and the maximum activities occurred at 2.5%. The immobilized protease showed a significant increase in activity (approximately 1.2–1.9 fold) compared to the soluble enzyme, especially at high NaCl concentrations (10–25%, w/v). They reported that the halotolerant nature of the immobilized protease makes the enzyme a promising candidate for various biotechnological applications.

**Figure 8:**
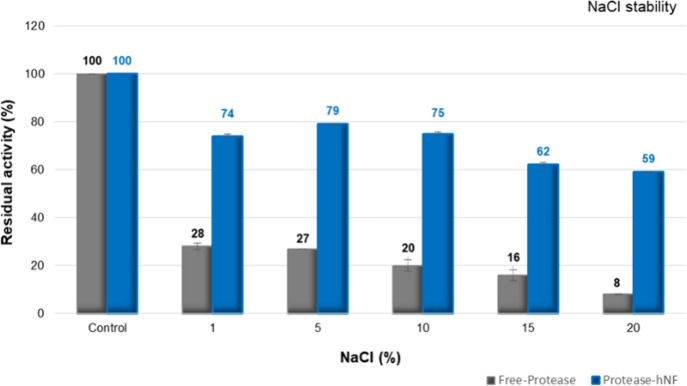
Effects of different NaCl concentrations on TNP13 free atP and atP@hNF.

### Determination of Hydrolysis Products (TLC)

In TLC analysis, it was determined by qualitative evaluation that free atP hydrolyzed casein to tyrosine, proline, lysine, aspartic acid, serine, and glycine **(** Figure 9 **). Arabacı and Karaytuğ** ^3^ incubated alkaline protease isolated from *B. pumilus* strain TNP93 with casein for 12 h. In the analysis, the enzyme degraded casein and released degradation products, such as arginine, histidine, cysteine, glycine, aspartic acid, and tyrosine. **Guleria and co-workers** ^34^ reported that the protease from *B. amyloliquifaciens* sp1 degraded casein to tyrosine and tryptophan in TLC.

**Figure 9:**
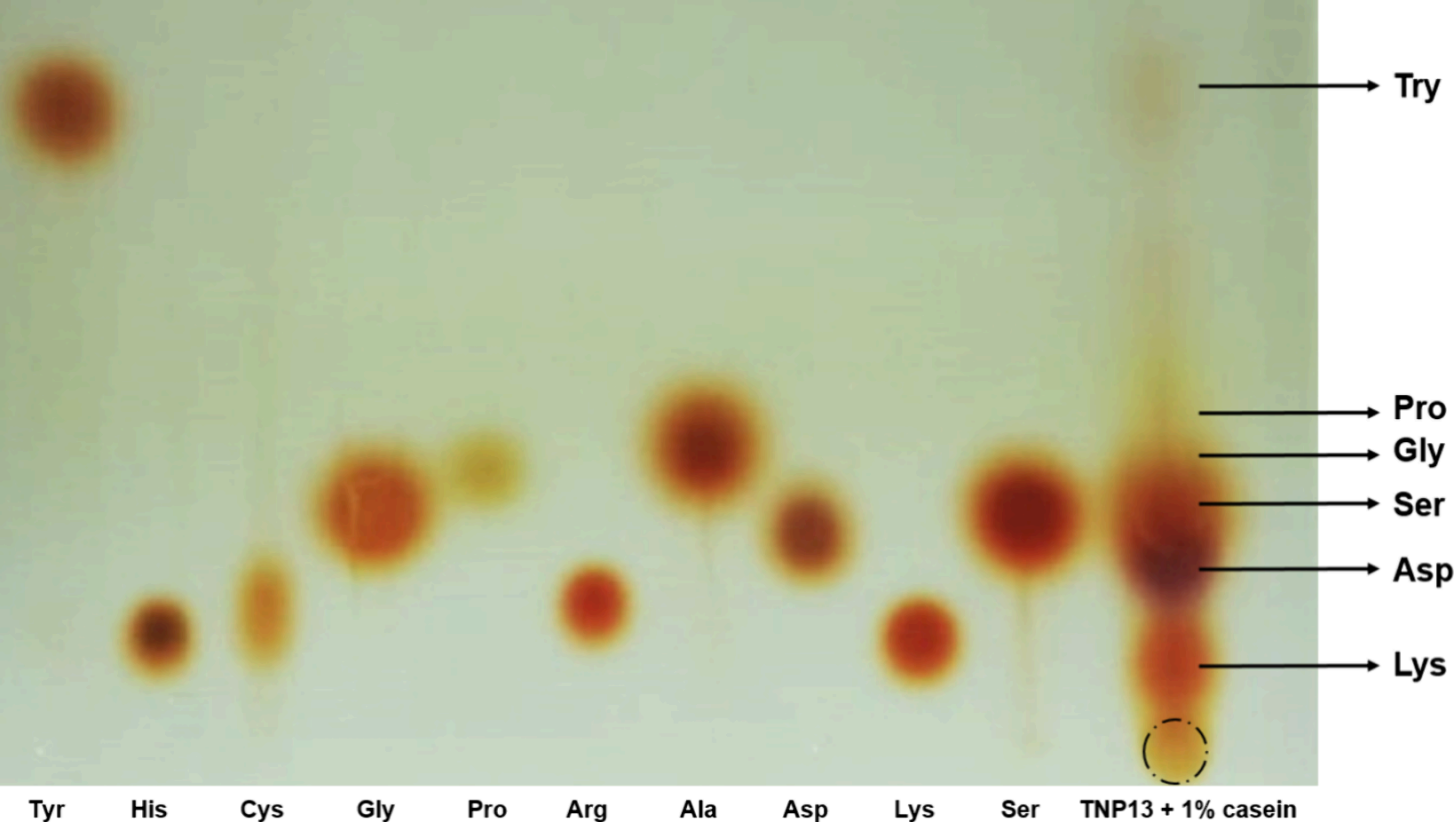
TLC of end products of TNP13 free atP-1% casein reaction (**Tyr:** Tyrosine, **His:** Histidine, **Cys:** Cysteine, **Gly:** Glycine, **Pro:** Proline, **Arg:** Arginine, **Ala:** Alanine, **Asp:** Aspartic acid, **Lys:** Lysine, **Ser:** Serine).

### Compatibility Analysis of atP@hNF with Commercial Detergents

The atP@hNF exhibited the highest compatibility with the ″Ariel″ brand liquid laundry detergent **(** Figure 10 **).** Washing performance analyses of both atP@hNF and free atP were conducted with “Ariel”. The enzyme maintained its stability with almost all liquid laundry detergents used in the test, and was compatible. **Gulmez and co-workers** ^44^ determined that the P-hNF was more stable than free proteinase K with different solid (OMO, ABC, ART, Ariel) and liquid (OMO, ALO, Perwoll, Ariel) commercial laundry detergents. The enzymes showed higher compatibility with liquid detergents than with solid ones. **Ibrahim and colleagues** ^47^ found that immobilized AK-R protease was more compatible with most tested commercial laundry detergents (Rex, X-Ttra, Ariel, Tide, Bonux, Persil) than its free form. They reported that the synthesized nanobiocatalyst is a promising candidate for laundry detergent formulations. **Badoei-dalfard and co-workers** ^14^ determined the compatibility of both free and nanoflower forms of collagenase with solid commercial detergents (1%) such as Barf, Dioxigeneh, Darya, Kaf, and Banoo. While free collagenase showed 80–105% activity against these detergents, immobilized collagenase (mcCNFs) exhibited higher activity than its free form. Immobilized collagenase was 30% and 38% more compatible than free collagenase against Dioxigeneh and Barf, respectively. Thus, they suggested free collagenase and mcCNFs could be used in detergents.

**Figure 10:**
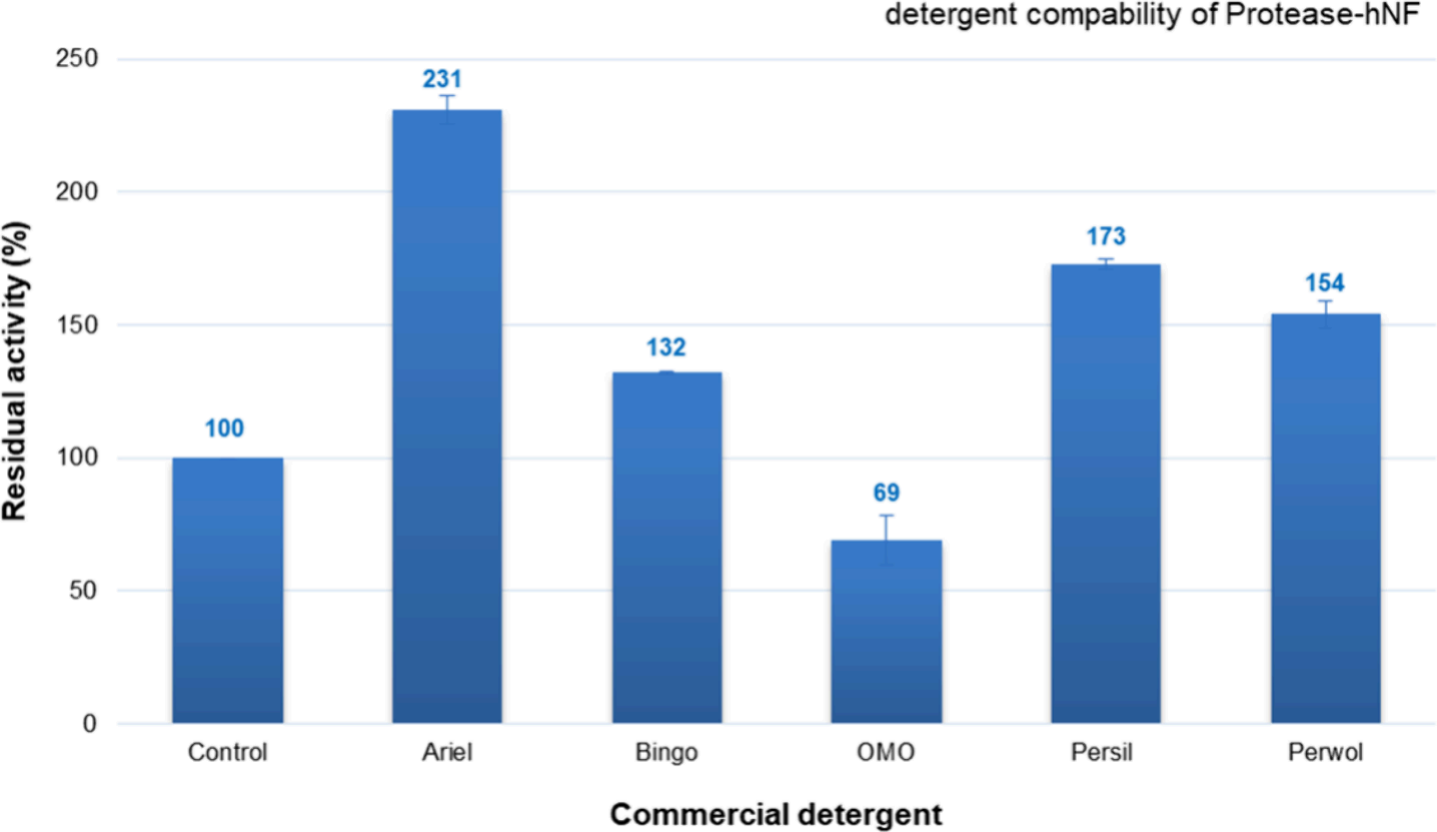
Compatibility analysis of atP@hNF with various commercial liquid detergents.

### Washing Performance Analysis

Adding microbial enzymes as biological additives to laundry detergent formulas increases the stain removal capacity of the detergent and reduces the chemical load in the detergent content. Therefore, it contributes to the reduction of the detergent amount required during washing. Developing environmentally friendly detergents is crucial to reducing synthetic chemicals such as surfactants, phosphates, chlorine, and bleaches. These substances can negatively impact human health, disrupt soil and water ecosystems, and threaten aquatic and terrestrial organisms.^58–61^ Incorporating enzymes like hNF with enhanced activity and stability into the laundry detergent formulation is essential for boosting the detergent’s cleaning efficacy, maintaining the integrity of the laundry fabric, and minimizing energy consumption.

In the washing tests performed on blood and milk stains on fabrics with code **EMPA 117 (** Figure 11A**)**, when free atP was used together with detergent (e) at 45 °C, it exhibited a better washing performance compared to untreated fabric (a), fabric washed only with tap water (b) and fabric washed only with free enzyme (d). For the same stain, washing with a combination of detergent and atP@hNF at 55 °C showed similar performance to washing with detergent and free enzyme. It also provided significantly better stain removal compared to washing with the enzyme alone (c).

**Figure 11:**
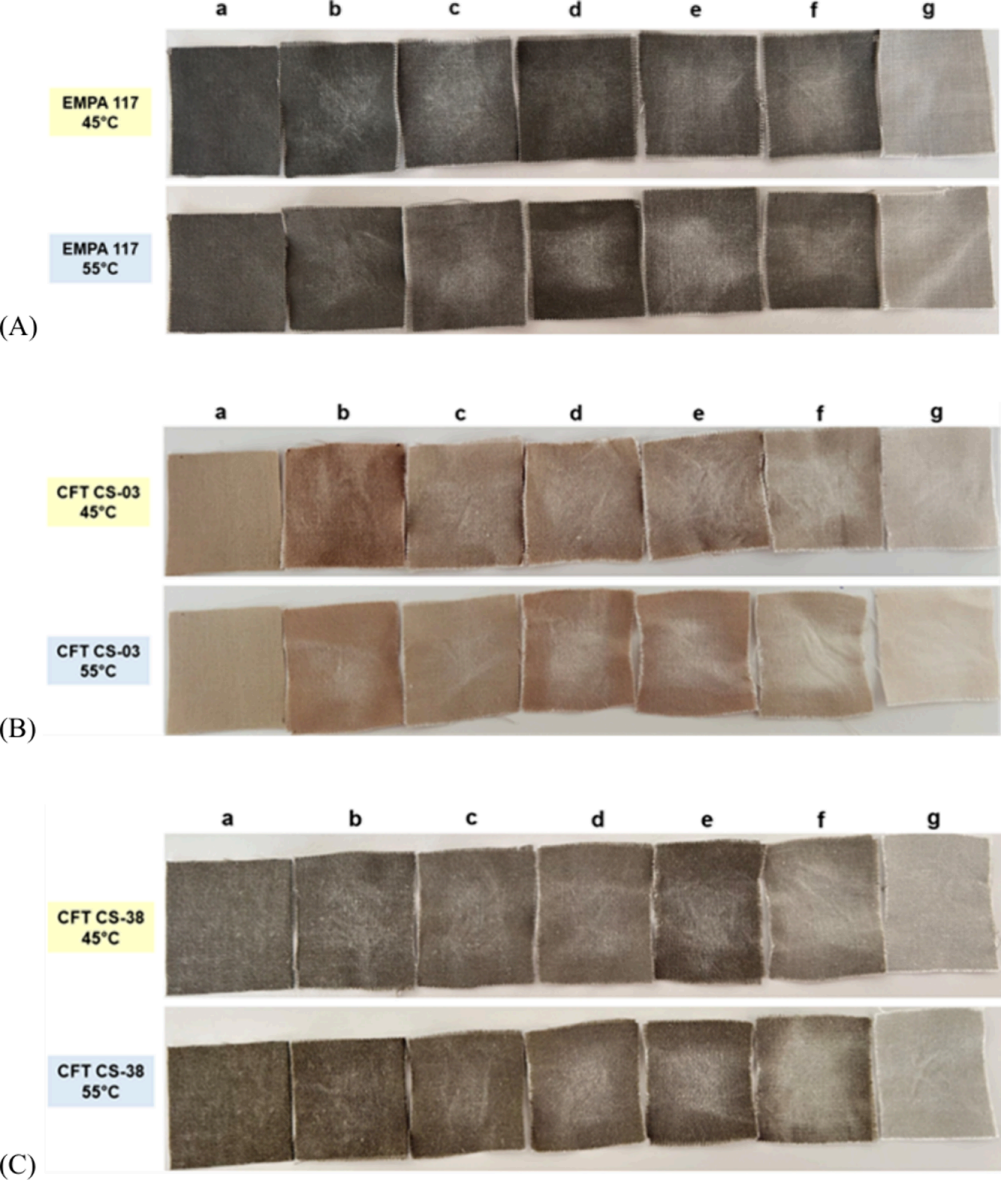
Washing performance analysis of free atP (45 °C) and atP@hNF (55 °C). (A) on EMPA 117 (blood/milk stain), (B) on CFT CS-03 (milk chocolate stain), (C) on CFT CS-38 (egg stain). [(a) untreated fabrics, (b) with tap water, (c) with detergent, (d) with enzyme (free atP, 45 °C; atP@hNF, 55 °C), (e) with detergent and enzyme (free atP, 45 °C; atP@hNF, 55 °C), (f) with commercial protease (washed at both 45 and 55 °C), (g) with detergent and commercial protease (washed at both 45 and 55 °C)].

In the wash performance analysis of milk chocolate stains on fabrics (coded **CFT CS-03**) **(** Figure 11B**)**, it is observed that when both free atP (45 °C) and atP@hNF (55 °C) were combined with the detergent (e), a better washing performance is exhibited compared to the stain removal on fabrics washed with only enzyme (d) and washed with detergent alone (c). However, no significant difference in stain removal performance was observed between atP@hNF and free atP.

In the washing analysis of egg stains on the fabrics coded **CFT CS-38 (** Figure 11C**)**, both washings performed with only free atP (45 °C) and only atP@hNF (55 °C) (d) exhibited a slightly better washing performance compared to the washings performed with enzyme alone (c) and tap water (b). When atP@hNF was used with detergent, better stain removal was achieved than washing with free enzyme-detergent.

In the washing performance analyses, TNP13 free atP and atP@hNF combined with commercial liquid laundry detergent showed better stain removal capacity than washing with tap water alone, detergent alone, and enzyme alone. atP@hNF was effective on all stains, especially EMPA 117 blood stains. Stain removal performances of commercial protease at both temperatures, in both enzyme-only and enzyme-detergent combination washings, were used as positive controls for comparison with washings conducted using free atP and atP@hNF. The stain removal capacity of atP@hNF was found to be quite effective, although not as much as the results from washes performed with commercial protease. Although the commercial enzyme shows better washing performance, our results demonstrate that immobilization enhances the washing efficiency of the atP@hNF compared to its free form. This improvement can be attributed to the increased stability and resistance of the enzyme in nanoflower form to denaturing detergent components. A key finding of this study is that the synthesized atP@hNF generally exhibits superior stain removal performance compared to free atP. The demonstration of atP@hNF’s effectiveness on stains when used in cowashing tests with commercial detergent indicates that it has great potential as a biological additive in laundry detergent formulas.

Additionally, we have not found any washing performance analysis using protease-hNF structures in our literature review. Although there are various research reports on synthesizing proteases and their derivatives as hybrid nanoflowers,^49,62–65^ no studies have reported using these nanomaterials in washing performance assessments. Therefore, our research is the first to evaluate the washing performance of proteases in the form of hybrid nanoflowers. This highlights the originality, novelty, and importance of our work.

## Conclusions

The present study characterizes the alkaline thermophilic protease isolated from the novel *Bacillus cereus* strain TNP13 and the organic–inorganic atP@hNF structures synthesized with Cu^2+^ metals using a single-step biomineralization process, highlighting their potential applications in the detergent industry. The atP@hNF structure was characterized using FESEM, EDX spectrum, elemental mapping, and FT-IR analyses. The enzymatic activity of atP@hNF increased by 8.56-fold compared to free atP. Literature with other free and immobilized proteases and proteolytic enzymes showed that both atP and atP@hNF demonstrated relatively high proteolytic activity. For example, alcalase-hNF exhibited a 1.57-fold increase in activity compared to free alcalase.^62^ Neutrase-hNFs showed 1.84-fold higher activity than free Neutrase.^66^ α-chymotrypsin-hNF showed a 3.04-fold activity over its free form.^67^ Notably, alkaline protease-Cu_3_(PO_4_)_2_.3H_2_O hybrid nanoflowers were reported to exhibit 9.27-fold and 10.94-fold higher activity compared to their respective free forms.^5,49^ These results indicate that atP@hNF is highly efficient in comparison with similar encapsulated hybrid enzymes reported in the literature.

Enzyme characterization studies were performed with both free atP and atP@hNF. Furthermore, analyses were carried out regarding the applicability of the atP@hNF in the detergent industry. The efficiency of the enzyme in removing the protein-based stains, such as blood, egg, and milk chocolate, from fabrics was assessed in washing analyses. The washing trials demonstrated that atP@hNF combined with detergent achieved superior stain removal compared to free atP alone. Detergent manufacturers prefer enzymes with high stain removal performance, and enzyme cost is an important consideration for them. Commercial enzymes are often expensive due to complex production processes. Therefore, the hybrid nanoflower form of the isolated protease can be produced at 30–83.27% lower cost than some commercial proteases. This may be a value-added product for detergent companies.

Applications regarding the use of enzyme-hNF in the detergent industry are quite limited in the literature, and commercial enzymes are preferred.^13,44,46^ In this context, the fact that washing performance analysis and compatibility test with commercial detergents were performed for the first time using bacterial alkaline protease-hNF that we synthesized in this study makes our research original. This highlights the value and the commercial importance of our study. Based on this, it is thought that the synthesized structures will be important potential candidates in the detergent industry, especially in laundry detergents. Additionally, we predict that various detergent formulations can be developed with the synthesized atP@hNF, and the detergent content can be standardized.

## Abbreviations

atP: alkaline thremophilic protease
atP@hNF: alkaline thremophilic protease-incorporated hybrid nanoflower
BSA: bovine serum albumin
EDX: energy-dispersive X-ray
FESEM: field emission scanning electron microscopy
FT-IR: Fourier transform infrared spectrometer
LB: Luria–Bertani
NCBI: National Center for Biotechnology Information
PBS: phosphate buffer saline solution
SDS-PAGE: sodium dodecyl-polyacrylamide gel electrophoresis
SMA: skim-milk agar
TCA: trichloroacetic acid
TLC: thin layer chromatography
3D: three-dimensional
XRD: X-ray diffractometer
